# Supplementary material for: COVID-19 vaccination uptake among healthcare workers in Ghana: A comprehensive analysis of knowledge, attitude, perceived vaccine effectiveness, and health belief model constructs
Source: PLOS Glob Public Health. 2024 May 2;4(5):e0002738. doi: 10.1371/journal.pgph.0002738 (PMC11065266; doi:10.1371/journal.pgph.0002738)
Supplement: S1 File — (PDF) [file pgph.0002738.s001.pdf]

Questionnaire

Section 1 (Socio-demographic characteristics)

Age: ____________________ Years

Sex: Male ◯ Female ◯

Occupation: (e.g. nurse, doctor, pharmacist, etc) ______________________________

Years of Practice ______________________________________ Years

Marital status: Married ◯ Not Married ◯

Working in frontline in the response to COVID-19 Yes ◯ No ◯

History of being diagnosed with COVID-19 Yes ◯ No ◯

Family member/friend ever diagnosed with COVID-19 Yes ◯ No ◯

Colleague ever diagnosed with COVID-19 Yes ◯ No ◯

Have you taken the COVID-19 vaccine?

Yes (One Dose) ◯

Yes (Two doses) ◯

No ◯

Section 2 (Knowledge about COVID-19 vaccination)

**Please tick ✓or circle the appropriate response**				
K1	Vaccines are effective in combating highly contagious Diseases	True	False	I Don’t Know
K2	Traditionally, vaccines create immunity by introducing a weak form of an infectious agent that allows the immune system to build a memory against this agent	True	False	I Don’t Know
K3	The RNA and DNA vaccines give our bodies the genetic code it needs to allow our immune system to produce the antigen on its own	True	False	I Don’t Know
K4	Covid-19 vaccines are being developed as quickly as possible, but they were required to receive the necessary regulatory licenses	True	False	I Don’t Know
K5	The flu vaccine protects against covid-19	True	False	I Don’t Know
K6	People with chronic diseases and elderly are more likely to have the disease and its complications, so they should get the vaccine	True	False	I Don’t Know
K7	Young people are healthy and therefore do not need to follow preventive measures and to get the vaccine in order to protect themselves against Covid-19	True	False	I Don’t Know
K8	Patients with chronic diseases like diabetes, hypertension and heart diseases are eligible to take the COVID-19 Vaccine	True	False	I Don’t Know
K9	Pregnant and lactating mothers are eligible to take the COVID-19 vaccine	True	False	I Don’t’ Know
K10	Until the readiness and the availability of COVID-19 vaccine, we cannot do anything to tackle the disease	True	False	I Don’t Know

Section 3 (Attitude about COVID-19 vaccination)

		**Please tick ✓or circle the appropriate response**				
A1	Do you think that many diseases prevented by vaccination are serious ones, mainly infectious?	Strongly disagree	Disagree	Neutral	Agree	Strongly Agree
A2	Do you think that the immunity acquired after contracting the disease is better than after vaccination?	Strongly disagree	Disagree	Neutral	Agree	Strongly Agree
A3	Do you think that it is better to wait for the next emerging vaccines than to get one of those developed in the first stage?	Strongly disagree	Disagree	Neutral	Agree	Strongly Agree
A4	Would you make a decision not to vaccinate for reasons other than illness or allergy?	Strongly disagree	Disagree	Neutral	Agree	Strongly Agree

A5	Would you delay getting vaccinated for reasons other than illness or allergy?	Strongly disagree	Disagree	Neutral	Agree	Strongly Agree
A6	Do you think that opinions on vaccines are primarily governed by the opinions and benefits of pharmaceutical companies?	Strongly Disagree	Disagree	Neutral	Agree	Strongly Agree

Section 4 (Perceived COVID-19 vaccine Effectiveness)

		**Please tick ✓or circle the appropriate response**				
PVE1	Do you think that vaccination against COVID-19 can protect you from contracting COVID-19?	Strongly Disagree	Disagree	Neutral	Agree	Strongly Agree
PVE2	Do you think that COVID-19 vaccination can prevent people from getting infected due to herd immunity?	Strongly Disagree	Disagree	Neutral	Agree	Strongly Agree
PVE3	Do you think mass vaccination against COVID-19 is justified?	Strongly Disagree	Disagree	Neutral	Agree	Strongly Agree

Section 5 (Health Belief Model Measures)

		**Perceived susceptibility to COVID-19**				
PSU1	I am susceptible of getting infected due to my occupational exposure	Strongly Disagree	Disagree	Neutral	Agree	Strongly Agree
PSU2	There is a great chance to get infected by COVID-19 in the next coming months especially during cold season	Strongly Disagree	Disagree	Neutral	Agree	Strongly Agree
PSU3	Healthy people can get COVID-19	Strongly Disagree	Disagree	Neutral	Agree	Strongly Agree
PSU4	My health status makes me more susceptible to contract COVID-19	Strongly Disagree	Disagree	Neutral	Agree	Strongly Agree
PSU4	I believe that I can protect myself against COVID-19 better than other people	Strongly Disagree	Disagree	Neutral	Agree	Strongly Agree
		**Perceived severity and seriousness**				
Sev1	Although for most people, COVID-19 causes mild illness, it makes some people very ill, and can be fatal	Strongly Disagree	Disagree	Neutral	Agree	Strongly Agree
Sev2	I think COVID-19 is more serious than any other Flu like illness	Strongly Disagree	Disagree	Neutral	Agree	Strongly Agree
Sev3	I will be very sick if I get COVID-19	Strongly Disagree	Disagree	Neutral	Agree	Strongly Agree
Sev4	If I get COVID-19, I might require hospitalization	Strongly Disagree	Disagree	Neutral	Agree	Strongly Agree

Sev5	If I get COVID-19, I might die	Strongly Disagree	Disagree	Neutral	Agree	Strongly Agree

5.3			**Perceived benefits of Vaccination**						
PB1	Vaccination is a good idea because it makes me feel less worried about catching COVID-19			Strongly Disagree	Disagree	Neutral	Agree	Strongly Agree	
PB2	Vaccination decreases my chance of getting COVID-19 or its complications			Strongly Disagree	Disagree	Neutral	Agree	Strongly Agree	
PB3	Vaccines are considered between the most tested and safe medical products			Strongly Disagree	Disagree	Neutral	Agree	Strongly Agree	
PB4	When I get vaccinated, I protect my patients, family and friends from infection			Strongly Disagree	Disagree	Neutral	Agree	Strongly Agree	
PB5	When I get vaccinated, the whole community benefits by preventing the spread of COVID-19			Strongly Disagree	Disagree	Neutral	Agree	Strongly Agree	
PB6	COVID-19 vaccination is an effective way to prevent and control COVID-19			Strongly Disagree	Disagree	Neutral	Agree	Strongly Agree	
PB7	High vaccination coverage globally is required to stop COVID-19 pandemic			Strongly Disagree	Disagree	Neutral	Agree	Strongly Agree	
			**Perceived barriers**						
PBA1	I am concerned that the vaccine is new and has not been used before			Strongly Disagree	Disagree	Neutral	Agree	Strongly Agree	
PBA2	I am concerned about the side effects of COVID-19 vaccine			Strongly Disagree	Disagree	Neutral	Agree	Strongly Agree	
PBA3	I am concerned about the efficacy of COVID-19 vaccine			Strongly Disagree	Disagree	Neutral	Agree	Strongly Agree	
PBA4	I am concerned about the safety of COVID-19 vaccine			Strongly Disagree	Disagree	Neutral	Agree	Strongly Agree	
PBA5	I am concerned about the accessibility of COVID-19 vaccines (geographical distribution of vaccination centers)			Strongly Disagree	Disagree	Neutral	Agree	Strongly Agree	
PBA6	I am concerned about the availability of COVID-19 vaccine in limited quantities for limited categories of the population			Strongly Disagree	Disagree	Neutral	Agree	Strongly Agree	
PBA7	I am concerned whether if the COVID-19 vaccine is allowed by my Religion			Strongly Disagree	Disagree	Neutral	Agree	Strongly Agree	
PBA8	I am concerned about the reliability of the manufacturer and the source of supply			Strongly Disagree	Disagree	Neutral	Agree	Strongly Agree	
PBA9	I am concerned about Ghana health system, and the strategy of distribution of the vaccines			Strongly Disagree	Disagree	Neutral	Agree	Strongly Agree	
PBA10	I am concerned about vaccine mode of administration (Injection)			Strongly Disagree	Disagree	Neutral	Agree	Strongly Agree	

PBA11	I am concerned about the number of doses of the vaccine that I have to take	Strongly Disagree	Disagree	Neutral	Agree	Strongly Agree
PBA12	I am concerned about the duration of immunity for the vaccine (how much time I will be protected)	Strongly Disagree	Disagree	Neutral	Agree	Strongly Agree

	**Cues of action**					
C1	Adequate and reliable information was necessary for me before taking the vaccine	Strongly Disagree	Disagree	Neutral	Agree	Strongly Agree
C2	I took the vaccine because it was recommended by the health facilities	Strongly Disagree	Disagree	Neutral	Agree	Strongly Agree
C3	I took the COVID-19 vaccine because it was recommended by a family member	Strongly Disagree	Disagree	Neutral	Agree	Strongly Agree
C4	I took the COVID-19 vaccine because it was recommended by the health authorities	Strongly Disagree	Disagree	Neutral	Agree	Strongly Agree
C5	I took the COVID-19 vaccine because it was widely recommended by the media	Strongly Disagree	Disagree	Neutral	Agree	Strongly Agree
C6	I took the COVID-19 vaccine after recommendations at my work	Strongly Disagree	Disagree	Neutral	Agree	Strongly Agree
C7	I took the COVID-19 vaccine because it was taken by many in the public	Strongly Disagree	Disagree	Neutral	Agree	Strongly Agree
C8	I took the vaccine because it was a requirement by my employers	Strongly Disagree	Disagree	Neutral	Agree	Strongly Agree

End!

Thank you
