# Supplementary material for: COVID-19 vaccination uptake among healthcare workers in Ghana: A comprehensive analysis of knowledge, attitude, perceived vaccine effectiveness, and health belief model constructs
Source: PLOS Glob Public Health. 2024 May 2;4(5):e0002738. doi: 10.1371/journal.pgph.0002738 (PMC11065266; doi:10.1371/journal.pgph.0002738)
Supplement: Attachment — Submitted filename: RESPONSE TO REVIEWERS.docx [file pgph.0002738.s003.docx]

20 Feb 2024

Editor: PLOS GLOBAL PUBLIC HEALTH

Dear Editor,

On behalf of my co-authors, we thank you for your valuable comments and the opportunity to submit a reviewed original manuscript entitled “**COVID-19 Vaccination Uptake among Healthcare Workers in Ghana: A Comprehensive Analysis of Knowledge, attitude, Perceived Vaccine effectiveness, and health belief model constructs**” for exclusive consideration for publication in PLOS Global Public Health.

We are thankful to the reviewers for their thorough review, and we believe that the manuscript has been significantly improved with their input.

Sincerely,

Please find our responses in red, highlighting how the manuscript has been modified in response to the reviewer's comments. Line numbers indicate the line numbers where changes have been made in the revised manuscript with Track Changes.

Reviewer # 1

Except for minor typographical errors and incomplete sentences observed, the manuscript is valid for PLOS Global Public Health acceptance for publication.

The manuscript has been proofread and all typographical errors and incomplete sentences have been corrected.

Reviewer # 2

Abstract

1. The abstract background looks lengthy; the authors could summarize it.

We have made changes to the introduction section of the abstract and reduced its length while retaining key background information necessary for understanding the study's objectives. This revision can be found on lines 12-16 of the revised manuscript.

2. Line 16 “Consequently, this study assessed uptake of COVID-19 vaccination and associated factors among healthcare workers in Ghana in the post-vaccine roll-out period.”.

What do the authors refer to as “this”?

In the statement "Consequently, this study assessed uptake of COVID-19 vaccination and associated factors among healthcare workers in Ghana in the post-vaccine roll-out period," the term "this" refers to the study itself—the research project that is being described in the abstract.

3. Line 19, healthcare workers were randomly selected? What type of random sampling approach was used?

We have revised the statement and specified the type of random sampling approach used. The new statement reads “256 healthcare workers were selected in Ayawaso West Municipality of Ghana using a stratified random sampling approach”. This revision is found on lines 20-22 of the revised manuscript.

4. Line 20-21 “Bivariable and Multivariable logistic regression was performed using IBM SPSS version 22 to identify predictors of vaccine uptake and a statistical significance was declared at p<0.05”. I suggest the authors change the verb “was” to were. Bivariable and multivariable tests are different. I suggest the sentence be revised. As it reads, it seems as if bivariable analysis can predict the study outcome.

We have revised the statement and replaced the verb “was” with the verb “were” in line 23 of the revised manuscript.

5. The results seem not to reflect the entirety of the study title. Where are the findings on perceived vaccine effectiveness?

The findings on perceived vaccine effectiveness are included in the results section of the abstract in lines 26-28 of the revised manuscript.

6. The methods section of the abstract can be written in detail.

We added more detail to the methods section of the abstract in lines 22-23 of the revised manuscript.

Introduction

1. The introductory section of the paper lacks information about COVID-19 in Ghana. It will be better if COVID information in Ghana is provided (e.g., case detection and strategies for prevention).

We have included information on case detection of COVID-19 in Ghana and strategies that were employed to contain the pandemic and prevent infection. This can be found in lines 62-68 of the revised manuscript.

2. Which reference style was used? Lines 73–64 seem to have a mixed referencing style. Authors should check and correct.

Thank you for catching this error in referencing. We have changed the referencing style from APA to Vancouver and the same has been consistently used in the manuscript. This edit can be found in line 90 of the revised manuscript.

3. The introduction also lacks information on knowledge, attitudes, and perceived effectiveness. The emphasis is laid on vaccine uptake. It is important that readers have a good understanding of the study outcomes in the introduction.

Thank you for this insight. We have included data on knowledge, attitude, and perceived vaccine effectiveness in the introduction. This can be found in lines 104-128 of the revised manuscript.

4. Line 65: Authors can furnish readers with current information; the May 2020 cases on COVID look outdated for a current paper. Authors can update.

We have updated the statement with the most recent published data on COVID-19 prevalence among healthcare workers. This can be found in lines 72-73.

Methodology

1. Line 168–170: The exclusion criteria are not clear. What explicitly defined participant exclusion in the study? Sick, disagreed to participate?

In lines 199-206, we have revised the manuscript to explicitly define participants' inclusion and exclusion criteria.

2. Line 186: How many health workers were from each health facility? This will help readers understand the proportionate allocation. Why only 14 in the 5 CHPS zones?

We have included the number of eligible participants that were from each facility in lines 221-236. Given the relatively smaller workforce size within CHPS zones compared to larger hospitals such as UGMC and Legon Hospital, a proportional allocation resulted in a smaller number of participants being allocated to CHPS zones.

3. Lines 187–188: “Secondly, stratification was done by profession type, and the sample size to be recruited from each profession was proportionally allocated.”. Stratification by profession type: how many groups were obtained in the stratification? The sampling approach needs major revision.

We have added information on the number of strata that resulted from the stratification process. We have further revised the sampling approach and added more information for clarity. The changes made can be found in lines 221-236

4. Which R command was used in the random selection?

In lines 234-237 we have added information to specify the command that we used in R to conduct the random selection of participants

5. Line 189: This statement could come earlier than line 185. I asked how you got to know the number of health workers in each facility without first contacting the Municipal health directorate or the human resource department of the facilities.

The statement in line 189 has been moved and appears earlier than the statement in line 185. This has now added clarity that we first obtained lists of workers from the human resource offices of each facility before determining the total number of workers at each facility.

6. Authors should be consistent with the use bivariable or bivariate.

We have consistently used the word bivariate.

7. Line 242-244 “Bivariate analysis was performed between socio-demographic characteristics and the outcome variable (uptake of COVID-19 vaccination) using the Chi-square test to determine potential confounding variables to include in logistic regression.”. I do not think chi-square test help in determining potential confounders, as stated, it tests an association between variables.

We have made changes to the statement and clarified that the Chi-square test was conducted to determine associations between socio-demographic factors and COVID-19 vaccine uptake. This can be found in lines 289-291

8. Line 249: What were the VIF values obtained to help readers know multicollinearity never existed in the predictor variables?

We have included the VIF ranges that were obtained in the analysis. Indeed, this will help readers know that multicollinearity never existed in our predictor variables. These changes can be found in lines 420 -422 under the results section.

9. Line 224: were the HCW who collected the data from the facilities. A brief description of them will be good.

Yes, the 3 research assistants were health workers from the 3 facilities (1 from each facility). A brief description of them is provided in lines 270-276.

Discussion

1. I do not think lines 411–418 are very relevant. The authors can clearly discuss the study findings.

We have deleted lines 411 to 418. This deletion can be found in lines 465-472 of the revised manuscript

2. What is the major difference between the heading in line 458 and the heading in line 496? This could be merged. Again this could come before the multivariate discussion.

We have merged the heading in line 496 with the heading in line 458 and this now appears before the discussion of the multivariate analysis (lines 538-542)

3.What could account for the disparity in association of the demographic factors and COVID-19 uptake in the comparison made with previous study (line 497-501). Authors could further explain.

We have added information to explain the possible reasons for the disparity in our findings with previous studies cited. This can be found on pages 542-551

4. Line 479: Adjusting for in adjusted model seems repetitive, adjusted model considers adjustment, hence no need to repeat.

We have edited the statement and deleted the words “in the adjusted model” in line 554.

5. 484–487: Repetition of Reference. The authors have referred and provided elaboration. This could be revised with references 80 and 83.

We have revised the references and removed the repeated references. This is in lines 562-563

Limitations

1. The health workers used in the data collection could have biased responses.

Healthcare workers were engaged as data collectors in our study, and this may introduce potential social desirability biases. Recognizing this, we took measures to minimize this bias. Completed questionnaires were submitted in sealed envelopes, preventing data collectors from viewing participants' responses. This ensured that data collection remained impartial and preserved respondent confidentiality. Despite these precautions, the possibility of biases cannot be entirely ruled out, and we have duly acknowledged this limitation in our study in lines 610-615

Reviewer # 3

It is true that author's findings provide data to an evidence-based formulation of vaccination strategies. Then, concluding that interventions to promote vaccination should target key determinants of vaccination uptake, such as attitude towards the vaccination and cues to action is reasonable. However, it would be great to propose a strategy for that o cite some reference.

We have proposed strategies for enhancing vaccine uptake in lines 578-605 under the section “Implications for policy and practice”.

Reviewer # 4

Introduction

1. The investigators have pointed out infection rates among doctors in Ghana. What was the infection rate among the other cadres of healthcare workers in Ghana. Was it higher than the regional and/or global infection rates? What was the mortality figures for healthcare workers in Ghana? How did it compare with the African or Global figures?

We have made changes to the introduction section in lines 76-85 and have included data on COVID-19 infection rate and case fatality rates among doctors, including other health cadres, particularly nurses, and how the infection rates and case fatality rates in Ghana compare with the African region and global figures.

2. You have mentioned inadequate data on the actual vaccination uptake among HCWs post-rollout. What do you mean by inadequate? Was there not even a single study about the actual hesitancy after the roll out?

We appreciate the reviewer's inquiry regarding the term 'inadequate' used to describe the available data on COVID-19 vaccination uptake among healthcare workers (HCWs) post-rollout. Upon careful consideration, we have revised the wording to more accurately reflect the absence of published studies specifically assessing the actual vaccination uptake among HCWs following the rollout of COVID-19 vaccines in Ghana. Therefore, we have replaced the term 'inadequate' with 'no studies were conducted' to convey this aspect. While several published studies focused on assessing the intention to vaccinate among HCWs prior to vaccine availability, our study uniquely evaluated vaccination uptake, defined as individuals who had been vaccinated. Unfortunately, despite an extensive review of the literature, we could not identify any published studies that directly assessed the acceptance of COVID-19 vaccination post-rollout among HCWs in Ghana. Therefore, our study fills an important gap in the literature by providing insights into the actual vaccination uptake among HCWs in the post-rollout period. This revision can be found in lines 126-129.

3. What was the vaccination acceptance rates reported in studies that you accessed? How does these figures compare with the region and global statistics?

Thank you for this insightful comment. We have included data on vaccine acceptance rates among healthcare workers in Ghana, from studies accessed, and how these rates compare with the rates reported in the African region. Lines 113-135

4. Although the authors identified the hesitancy among HCWs, they didn’t delve deeper into the reasons behind this hesitancy. Therefore, exploring the factors contributing to vaccine hesitancy among HCWs could enrich the discussion.

We have added information in the introduction on factors contributing to vaccine hesitancy. This can be found in lines 118-125

5. The conceptual framework is not provided and should be included.

The conceptual framework is part of the manuscript (Fig 1) and narration for the framework is in lines 144-173

Methodology

1. The duration of data collection has been mentioned but it might be beneficial to provide reasoning for the specific timeframe chosen, especially concerning any potential fluctuations or significant events that could affect participants' perceptions or behaviors during the period mentioned.

We appreciate the reviewer's suggestion regarding providing reasoning for the specific timeframe chosen for data collection. The data collection period coincided with the academic calendar, aligning with the timeline designated for conducting the study. We therefore did not have any specific rationale for choosing the data collection period. We, however, acknowledge that providing further rationale for this timeframe could offer valuable insights into potential fluctuations or significant events that may have influenced participants' perceptions or behaviors.

2. Some of the inclusion and exclusion criteria pointed out are not well thought and should be rewritten or moved to their right section. For instance, consenting to a study should be moved to ethical consideration section.

We have revised the inclusion and exclusion criteria and further moved consenting to the ethical consideration section. These changes can be found in lines 199-206

3. The definition of COVID-19 vaccine uptake is clear; however, specifying the types of vaccines considered might add clarity to the outcome measure, given the availability of different vaccines at different times.

Our primary focus was on assessing whether participants had received any type of COVID-19 vaccine rather than the specific type received. Therefore, while we acknowledge the availability of different vaccines over time, our outcome measure centered on the overall uptake of COVID-19 vaccination, regardless of the specific vaccine type.

4. The section briefly describes the statistical analysis plan, including bivariate and multivariable logistic regression. Further details on the specific variables included in the model, handling of missing data, and assumptions made could enhance the rigor of the analysis explanation.

We manually inspected all returned questionnaires for completeness and only completed questionnaires were included in the analysis (lines 285-286). Variables included in the model are described in lines 426-437 and can be found in Table 7.

5. What informed the choice of statistical significance for univariable regression analysis which was set at p<0.2?

A cut-off p-value of <0.2 ensured that our multivariable regression analysis included all potentially important predictive variables. We have added information to explain the rationale and further added a reference. This can be found in lines 294-296.

6. The study adequately addresses ethical considerations, including approvals and participant consent. However, detailing steps taken to ensure data confidentiality and participant privacy during data storage and analysis would further strengthen the ethical assurance.

We have clarified in lines 308-309 on how we safeguarded participants' confidentiality. Furthermore, we have added information to clarify how participants’ privacy was ensured during data storage and analysis (lines 310-313).

7. The authors should consider adding recall bias or social desirability bias in self-reported responses as part of the limitations.

Social desirability bias is one of the limitations that was recognized in our study. This can be found in lines 611-612. While recall bias could be a potential limitation in our study, considering that participants were asked to recall whether they had received the vaccination or not, we regarded it less problematic due to the recent and salient nature of COVID-19 vaccination, making it less susceptible to recall bias. Additionally, participants were likely to have accurate recollection of their vaccination status, as it was considered a significant aspect of their professional and personal lives.

Results

1. Despite high overall awareness, specific gaps exist, particularly regarding the eligibility of pregnant and lactating mothers for COVID-19 vaccination. How did you handle these parameters?

Thank you for bringing up this important point. While our analysis predominantly focused on the overall knowledge score regarding COVID-19 vaccination, we acknowledge the specific gap identified in participants' awareness regarding the eligibility of pregnant and lactating mothers for vaccination. We have discussed this gap and included a specific recommendation to address it in lines 496-500.

2. While the study identified various barriers to vaccination, deeper exploration of their impact on vaccination behavior could enhance the practical implications of the findings.

Thank you for your additional guidance. As our study primarily employed quantitative methods to identify barriers to vaccination, we recognize the importance of complementing these findings with qualitative research to gain a deeper understanding of their impact on vaccination behavior. We have therefore included a recommendation for qualitative studies to explore in-depth the barriers to vaccination identified in this study. This recommendation can be found in lines 591-594.

Discussion

1. What are the implications of your findings?

We have included a section on the implications of our findings in lines 578-605

Reviewer # 5

Introduction

1. In the introduction, the data regarding the number of cases and deaths is from early 2023. Considering the publication is now, I suggest updating it with the latest data from 2024 or simply updating the data until the data collection in August 2023 (to provide contextualized information).

In lines 55-58, we have updated the latest data from January 2024 on COVID-19 infection rates and deaths globally.

2. In line 57, I believe a semicolon should not be there; I suggest leaving the sentence with just commas.

Thank you for correcting this error. We deleted the semicolon and replaced it with a comma.

3. In line 156, the word "healthcare" is in lowercase and should be capitalized.

We found the error in line 166 and we have capitalized the word “healthcare” and changed it to “Healthcare” in line 199 of the revised manuscript

Methodology

1. Figure 1 does not look very appealing. I think it lacks color, and the lines appear crooked. It is a super interesting figure that describes crucial information to understand the entire reasoning of the article. I believe it can be made more didactic and attractive to the reader.

we have modified Figure 1 to enhance its visual appeal and clarity.

Results

1. In the results section, some information is presented with the numeral itself without parentheses or connecting words ("The majority of participants 234 (91.4%) were aware"). Personally, I am not fond of this sentence structure, but it is at the authors' discretion. I would suggest a more elaborate construction. This repeats several times ("More than three-quarters 223 (87.1%) of participants"; "About one-third 77 (30.1%) of the participants were nurses"; "Almost equal numbers of participants were recruited from UGMC 123 (48.0%) and Legon Hospital 120 (46.9%)”.

The chosen sentence structure, which presents numerical values directly followed by percentages was purposefully employed to provide additional information on percentages. By including the numerical values alongside the percentages in this manner, we aimed to offer readers a more comprehensive understanding of the data presented.
